# A Rare Case of Leuconostoc pseudomesenteroides Bacteremia and Refractory Septic Shock

**DOI:** 10.7759/cureus.38312

**Published:** 2023-04-29

**Authors:** Mike Ghobrial, Mohamed Ibrahim, Spencer G Streit, Peter P Staiano, Vandana Seeram

**Affiliations:** 1 Internal Medicine, University of Florida College of Medicine – Jacksonville, Jacksonville, USA; 2 College of Osteopathic Medicine, Lake Erie College of Osteopathic Medicine, Bradenton, USA; 3 Pulmonary and Critical Care Medicine, University of Florida College of Medicine – Jacksonville, Jacksonville, USA

**Keywords:** gram-positive bacteria, vancomycin resistant, bacteremia, refractory septic shock, leuconostoc pseudomesenteroides

## Abstract

*Leuconostoc* species are rare causes of bacteremia and are commonly mistaken for *Streptococcus *species. Due to their inherent resistance to commonly used drugs, they are often overlooked resulting in poor and sometimes lethal outcomes. While there are not many reported cases of this bacterial infection, *Leuconostoc *species are important to consider when faced with a highly drug-resistant bacterial strain. We present here a case of a 29-year-old male who presented with an out-of-hospital cardiac arrest, initially resuscitated but ultimately succumbing to his illness. This is a unique case in which our patient was subsequently found to have a rare bloodstream infection with *Leuconostoc pseudomesenteroides.*

## Introduction

*Leuconostoc* species (spp.) are gram-positive, catalase-negative, and lactic acid-producing cocci that were previously thought to have a low pathological profile [1]. They are typically used in various parts of the food industry as starter cultures and can also be found on different plants such as leafy vegetables and sugar cane [2]. Although they are not part of the normal human microbiome, they are occasionally found in the female vagina and some stool samples [1]. Despite an overall paucity in the literature, these organisms are becoming increasingly recognized for their pathogenic potential due to an inherent resistance to vancomycin (VCM) and other glycopeptide antibiotics like teicoplanin [1]. This innate resistance comes from the lack of a target protein on the cell wall of this species [1]. Belonging to the *Streptococcaceae* family, this organism is often mistaken for similarly structured, VCM-sensitive bacteria such as *Lactobacillus*, *Streptococcus*, and *Enterococcus* [3,4]. *Leuconostoc* spp. usually present in the immunocompromised patient population, and as to be described in this case, may have poor and fatal outcomes [1]. Here we present a rare case of refractory septic shock in the setting of *Leuconostoc pseudomesenteroides* bacteremia.

## Case presentation

A 29-year-old male with a past medical history of poorly controlled type 1 diabetes mellitus, chronic hepatitis C, and a history of intravenous drug use was recently hospitalized for methicillin-resistant *Staphylococcus aureus* (MRSA) bacteremia complicated by infective endocarditis, in which he completed only 12 days of intravenous (IV) vancomycin due to leaving against medical advice. He was prescribed linezolid at that time to take with him; however, it remained unknown if he completed his antibiotic course. He presented to the emergency department one month later after being found down unresponsive in an outdoor park. He was pulseless with an unknown downtime, in which emergency medical services initiated resuscitative efforts and continued until arrival to the hospital. In the emergency department, return of spontaneous circulation (ROSC) was achieved. Following ROSC, he was in refractory shock, requiring several vasoactive agents including norepinephrine, epinephrine, and vasopressin. He was subsequently intubated and initiated on empiric IV antibiotics that included vancomycin, cefepime, and metronidazole. He was hypothermic at 94.5°F, with tachycardia at 120 bpm, and on physical examination, he remained unresponsive with dilated pupils and diffuse coarse breath sounds bilaterally. A bedside transthoracic echocardiogram was performed revealing a left ventricular ejection fraction of 45%, no evidence of valvular pathology, and no pericardial or pleural effusions.

**Figure 1:**
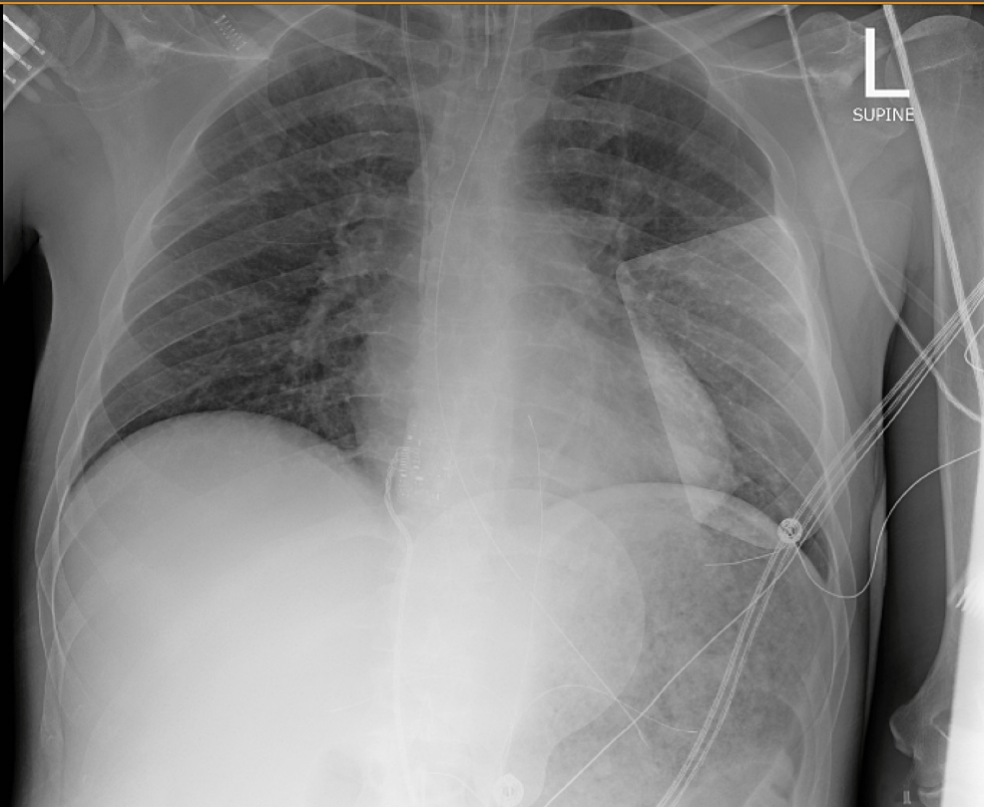
Chest radiograph in the anterior-posterior (AP) view showing a subtle opacity in the right upper lobe

Laboratory testing revealed a white blood cell count of 31.4 x 10^3^/uL with 14.2% bandemia, lactic acid 15.7 mmol/L, arterial blood gas notable for severe metabolic acidosis with a pH of 6.94, and a bicarbonate level of 10 mmol/L. Further analysis revealed elevated transaminases, an acute kidney injury, thrombocytopenia, and elevated coagulation parameters consistent with disseminated intravascular coagulation (DIC) in the setting of septic shock. Laboratory test results are summarized in Table 1. Chest x-ray showed subtle right upper lobe patchy infiltrates (Figure 1). A bicarbonate infusion was started due to refractory metabolic acidosis. His preliminary blood culture demonstrated gram-positive cocci in pairs or chains suggestive of *Streptococcus*. Despite all aggressive interventions, he continued to deteriorate and subsequently suffered a pulseless electrical activity (PEA) arrest with an unsuccessful attempt at resuscitation. After the patient succumbed to the overwhelming refractory shock, final blood culture results speciated as *Leuconostoc pseudomesenteroides*.

**Table 1:** Laboratory values on admission and at four hours post-admission INR: international normalized ratio, LDH: lactate dehydrogenase, pH: potential of hydrogen, PCO_2_: partial pressure of carbon dioxide, AST: aspartate aminotransferase, ALT: alanine aminotransferase, ALP: alkaline phosphatase

Laboratory parameters	Reference range	Admission values	4 hours later
Sodium (mmol/L)	135-145	126	133
Potassium (mmol/L)	3.3-4.6	6.8	5.2
Urea (mg/dL)	6-22	54	60
Creatinine (mg/dL)	0.67-1.17	1.11	1.50
Bicarbonate (mmol/L)	21-29	10	14
Phosphorus (mg/dL)	2.5-4.5	15.4	14.5
White cell count (per mm^3^)	4,500-11,000	24,000	31,400
Bands (%)	0-10%	5.2%	14.2%
Procalcitonin (ng/mL)	<0.05	N/A	1.44
Hemoglobin (g/dL)	14-18	10.2	9.0
Platelet count (per mm^3^)	140,000-440,000	265,000	102,000
INR	0.8-1.1	1.4	3.8
Protime (seconds)	9.4-12.5	15.5	42.5
Fibrinogen (mg/dL)	186-461	N/a	70
LDH (U/L)	126-266	N/a	5300
Arterial pH	7.35-7.45	6.94	6.99
Arterial PCO_2 _(mmHg)	35-45	37	51
Lactic acid (mmol/L)	0.7-2.7	15.7	>20
AST (U/L)	14-33	1099	2278
ALT (U/L)	10-42	539	834
ALP (U/L)	40-129	211	551

## Discussion

This case report describes the pathogenicity and virulence of *Leuconostoc pseudomesenteroides*, which is rarely seen in the clinical setting. *Leuconostoc* species are gram-positive, catalase-negative, lactic acid-producing cocci that are ubiquitous, commonly being found in plants, dairy and food products, and are typically of low pathogenic potential in humans [1]. As a result, they are most commonly found in the immunocompromised population and are usually implicated with other infections [5]. While the first case of *Leuconostoc* infection in humans was reported by Buu-Hoï et al. in 1985, the prevalence and incidence of this organism in the hospital setting is unknown [6]. This is largely due to the misidentification of *Leuconostoc* species for other common gram-positive bacteria such as *Streptococcus* and *Enterococcus *due to similarities in their phenotypic resemblance [5]. Several distinguishing laboratory characteristics aiding in the differentiation of these organisms have been identified. According to Bernaldo de Quirós et al., *Leuconostoc* species are identified by the following criteria: gram-positive cocci, vancomycin resistance, a lack of catalase production, negative Voges-Proskauer test, production of gas from glucose in de Man Rogosa and Sharpe (MRS) agar, a lack of arginine deamination, and negative reactions to pyrrolidonyl arylamidase (PYR) [7]. *Leuconostoc* species are naturally resistant to glycopeptides because their pentapeptide cell wall precursors end in an alanine-lactate depsipeptide rather than the traditional vancomycin binding site dipeptide of alanine-alanine [8].

Although the total incidence of *Leuconostoc* bacteremia remains uncertain, Kaboré et al. presented the prevalence of *Leuconostoc* species in endodontic infections to be between 1.2% and 2.9% [9]. This is important as it is widely known that dental infections and endodontic work can cause systemic infections such as endocarditis, which could ultimately lead to sepsis and death [9]. As we see in our patient, he was admitted to the hospital one month prior for infective endocarditis and although he was a long-term intravenous drug user, it is important to note that his lack of hygiene could have also allowed for an underlying *Leuconostoc* infection to develop. Due to the immunocompromised state of most patients reported with *Leuconostoc* infections, it is important to also note its potential for nosocomial spread. In 2008, Bou et al. described the outbreak of *Leuconostoc* species in the Juan Canalejo Hospital (northwest Spain) in 48 patients [5]. The study concluded that the *Leuconostoc* organism is an important emerging pathogen to consider as the nosocomial infection in patients who have underlying diseases.

It is a routine clinical practice within the United States to initiate vancomycin as part of a broad-spectrum antibiotic regimen when a patient is identified in septic shock. However, patients who do not respond to initial resuscitation efforts, including after starting VCM, should be considered for resistant organisms and switched to an alternative antibiotic that will cover vancomycin-resistant pathogens. If recognized early enough, these organisms are typically susceptible to penicillin G, ampicillin, clindamycin, carbapenem, and/or aminoglycosides [1]. However, a study by Ino et al. in 2016 showed a high minimum inhibitory concentration (MIC) to meropenem, suggesting that carbapenem-resistant isolates could exist [1]. As such, the mainstay of treatment is currently penicillin monotherapy or in combination with an aminoglycoside. Other therapeutic options include clindamycin and carbapenems, but therapy should be tailored based on lab sensitivities.

Due to the nonspecific symptoms and similarities between *Leuconostoc* bacteremia and other causes of sepsis, we stress a systematic workup in patients who present with signs of shock and no response to traditional broad-spectrum empiric IV antibiotics such as vancomycin. While this antibiotic is most commonly the first-line treatment due to its encompassing coverage of gram-positive bacteria, penicillin G and/or aminoglycosides should be considered when dealing with a vancomycin-resistant bacterial strain. Our case study is not without limitations as the primary source of the infection remains unknown. As mentioned above, *Leuconostoc* species can contaminate the oral flora and be the cause of dental infections that are known to be the basis of potential episodes of infective endocarditis, which our patient was diagnosed with in his previous hospital visit. However, he was also an intravenous drug user and a recurring patient in the hospital, as such a nosocomial source could have also been the culprit for the bacteremia. We urge clinicians to think of *Leuconostoc* species on their differential when faced with gram-positive, vancomycin-resistant bacteria unresponsive to conventional protocols.

## Conclusions

*Leuconostoc* infections are a rare cause of bacteremia but should be considered as a causative pathogen when vancomycin-resistant, gram-positive cocci are discovered on blood cultures. While it is mostly described in immunosuppressed patients, it should also be a differential diagnosis in immunocompetent patients who have a history of recent antibiotic exposure, intravenous drug use, infective endocarditis, use of parenteral/enteral nutrition, or recent dental work. Our patient rapidly succumbed to septic shock due to *Leuconostoc pseudomesenteroides* which was resistant to vancomycin. Although rare, it is important to consider intrinsically vancomycin-resistant bacteria such as *L. pseudomesenteroides* in patients with refractory septic shock despite the broad empiric antibiotic coverage with the typically utilized agents.
